# Two retailer–supplier supply chain models with default risk under trade credit policy

**DOI:** 10.1186/s40064-016-3346-3

**Published:** 2016-10-06

**Authors:** Chengfeng Wu, Qiuhong Zhao

**Affiliations:** 1 School of Economics and Management, Qingdao University of Science & Technology, 99 Songling Rd. Laoshan Dist., Qingdao, 266061 People’s Republic of China; 2 School of Economics and Management, Beihang University, 37 Xueyuan Rd., Haidian Dist., Beijing, 100191 People’s Republic of China

**Keywords:** Supply chain, Trade credit, Default risk, Supplier-Stackelberg game, Nash game

## Abstract

The purpose of the paper is to formulate two uncooperative replenishment models with demand and default risk which are the functions of the trade credit period, i.e., a Nash equilibrium model and a supplier-Stackelberg model. Firstly, we present the optimal results of decentralized decision and centralized decision without trade credit. Secondly, we derive the existence and uniqueness conditions of the optimal solutions under the two games, respectively. Moreover, we present a set of theorems and corollary to determine the optimal solutions. Finally, we provide an example and sensitivity analysis to illustrate the proposed strategy and optimal solutions. Sensitivity analysis reveals that the total profits of supply chain under the two games both are better than the results under the centralized decision only if the optimal trade credit period isn’t too short. It also reveals that the size of trade credit period, demand, retailer’s profit and supplier’s profit have strong relationship with the increasing demand coefficient, wholesale price, default risk coefficient and production cost. The major contribution of the paper is that we comprehensively compare between the results of decentralized decision and centralized decision without trade credit, Nash equilibrium and supplier-Stackelberg models with trade credit, and obtain some interesting managerial insights and practical implications.

## Background

In the past 100 years, a huge of extensions of the traditional Economic Order Quantity (EOQ) model has been proposed by lots of researchers. Recently, the International Journal of Production Economics published a special issue “Celebrating a century of the economic order quantity model in honor of Ford Whitman Harris”. Among them, Andriolo et al. (2014) and Glock et al. (2014) respectively adopted different methodologies to analysis the evolution and main streams of these research emerged from Harris’ seminal lot size during 100 years of history and proposed a new research opportunities for future research, such as, sustainability issue and cash flows. Latest works include those by Battini et al. (2014), and Marchi et al. (2016), among others.

One of most important extensions is that incorporating trade credit into the EOQ. It assumes that supplier offers retailer/buyer a permissible delay in payments (trade credit period). The account is not settled during trade credit period and there is no interest charge. In fact, as short-term financing tool, trade credit is widely implemented in fierce competitive circumstance and has important influence on inventory holding cost (Azzi et al. 2014).

Goyal (1985) first fully analysis the impact of fixed permissible delay in payments on the retailer’s ordering decision. Since then, the effect of fixed trade credit on the replenishment policy has been studied in extensive literatures. For instance, Aggarwal and Jaggi (1995) extended Goyal’s model (1985) to consider exponentially deteriorating items. Teng (2002) further established an easy analytical closed-form solution about Goyal’s model (1985) by considering the difference between the purchase cost and the retail price. Furthermore, Huang (2003) proposed a brand new inventory model under two levels of trade credit where the manufacturer offers trade credit to the retailer, and the retailer also offers his or her customer partial trade credit.

Teng et al. (2012a, b) extended the constant demand to a linear increasing demand under trade credit. Wu and Zhao (2015a) recently established an EOQ model with a constant deterioration rate, a current inventory-dependent and linearly increasing time-varying demand under trade credit and presented some fundamental theoretical results. Lots of related articles can be seen in Khouja and Mehrez (1996), Chu et al. (1998), Chang and Dye (2001), Teng and Chang (2009), Jain and Aggarwal (2012), Chung (2013), Zhou et al. (2013), Chen and Teng (2014), Ouyang et al. (2014), among others. However, most of these models assumed that a trade credit period is fixed parameter and the retailer sets up its own strategy only from its individual perspective. Elaborating on this subject, Chang et al. (2008), Seifert et al. (2013), and Molamohamadi et al. (2014) conducted comprehensive literature reviews of different model settings.

On the other hand, like quantity discount, price discount, etc., as a profit transfer means, trade credit has been deeply studied in supply chain coordination. For instance, Yang and Wee (2006) presented a collaborative model for deteriorating items with price-sensitive demand and finite replenishment rate under trade credit and proposed a negotiation factor to share the additional profit between the vendor and buyer. Sarmah et al. (2008) considered the issue of coordination with trade credit term in a single supplier and multiple heterogeneous retailers at same replenishment cycle time. Wu and Zhao (2014a) recently established a collaborative model under trade credit for inventory-dependent and time-varying demand during the finite planning horizon. Other related articles can be seen in Jaber and Osman (2006), Chen and Kang (2007), Huang et al. (2010), Chan et al. (2010), Krichen et al. (2011), Teng et al. (2012a, b), Hsu and Hsu (2013), Wu and Zhao (2014b), Glock and Kim (2015), and Marchi et al. (2016), among others. These papers assumed that trade credit is a decision variable and coordination mechanism, and discussed the effect of trade credit in coordinating supply chain for different settings.

However, how to determinate an optimal trade credit period for the supplier has been received limited attention for a long time. Although, Kim et al. (1995) first proposed a strategy to determine the optimal trade credit period for supplier and the optimal pricing for the retailer in supplier-Stackelberg game. And then, Abad and Jaggi (2003) extended the model of Kim et al. (1995). The two literatures did not arouse scholars’ attention at that time. The question can be really solved until Teng and Lou (2012) proposed the demand rate is an increasing function of the trade credit period (a decision variable). Although, Jaggi et al. (2008) first assumed that trade credit period has a positive impact on demand and set up a polynomial function, where trade credit period is still given parameter. Of course, other researcher also proposed inventory model with demand rate is dependent on the trade credit period, such as, Ho (2011), Giri and Maiti (2013), the trade credit period is constant.

At present, determining optimal trade credit period is being more and more attention from researchers. There are two main research views, one is trade credit provider perspective, and other is game perspective. From trade credit provider perspective, for instance, Lou and Wang (2012) extended Teng and Lou’s model (2012) to establish an EOQ model to derive optimal trade credit period and lot size simultaneously. But, in their model, they didn’t concern the retailer’s benefit and an additional capital opportunity cost the supplier burdens for offering trade credit. Recently, Teng et al. (2014) extended Lou and Wang’s model (2012) to consider learning curve phenomenon and the loss of capital opportunity during the delay payment period. Dye and Yang (2015) further extended Lou and Wang’s model (2012) to include cases with partial backorder and the supplier’s opportunity cost and two carbon emission constraints. Chen and Teng (2015) recently extended Teng and Lou’s model (2012) to consider time-varying deteriorating items and default risk rates under two levels of trade credit by discounted cash flow analysis. Other prominent and latest works include those by Wang et al. (2014), Wu et al. (2014), and Shah and Cárdenas-Barrón (2015), among others.

Determining optimal trade credit period from the game perspective is becoming concerned. Only a few corresponding articles may be found in latest literatures. Zhou et al. (2012) established an uncooperative inventory model for items with stock-dependent demand where the retailer has limited displayed-shelf space, and optimized the trade credit period in a two-echelon supply chain. Zhou and Zhou (2013) investigated two trade credit scenarios, i.e., unconditional and conditional trade credit, and discussed how the supplier sets up trade credit period to minimize his or her cost under supplier-Stackelberg game in a two-echelon supply chain. Based on the models of Zhou and Zhou (2013) and Teng et al. (2014), Wu and Zhao (2015b) established an uncooperative replenishment model with time-varying demand and time-varying price and learning curve phenomenon under finite planning horizon and supplier-Stackelberg game. However, these cited references do not consider the effect of trade credit period on market demand and default risk.

Additionally, based on the achievements of Teng and Lou (2012) and Lou and Wang (2012), Chern et al. (2013) recently established a vendor–buyer Stackelberg equilibrium model with compounded interest rate and relaxing lot-for lot replenishment policy, and derived the vendor’s optimal ordering policy and trade credit period. Chern et al. (2014) extended the model of Chern et al. (2013) to establish a vendor–buyer supply chain model in Nash game. But the two references ignored the results of decentralized decision and centralized decision without trade credit, and didn’t compare with the results of Nash equilibrium and supplier-Stackelberg models with trade credit in detail.

In this paper, we discuss about two retailer–supplier uncooperative replenishment models with trade credit where the demand and default risk are liked to trade credit period, i.e., a Nash equilibrium model and a supplier-Stackelberg model. We comprehensively compare between the results of decentralized decision and centralized decision without trade credit, and Nash game and supplier-Stackelberg models with trade credit. We distinguish the impact of trade credit period on the demand and default risk to observe two parties’ profit and behavior.

The remainder of the paper is organized as follows. In “Assumptions and notation” section, assumptions and notation are presented. In “Mathematical formulation of the model without trade credit” section, we present the decentralized and centralized inventory models without trade credit. In “Mathematical formulation of the models with trade credit” section, we derive uncooperative supply chain inventory models with trade credit in Nash game and supplier-Stackelberg game, respectively. In “Numerical examples and analysis” section, we present a numerical example and sensitivity analysis, and propose important conclusions on managerial phenomena. The last section summarizes the paper’s findings and suggests areas for future research.

## Assumptions and notation

The following assumptions and notation are used throughout the paper. Some assumptions and notation will be presented later when they are needed.

### Assumptions

(i) Permissible delay in payments or trade credit attracts new buyers who consider it to be a type of price reduction. According to the previous literatures, such as that by Teng and Lou (2012), Lou and Wang (2012), Chern et al. (2013), and Teng et al. (2014), among other authors, demand rate is assumed to be a polynomial or exponential function of the trade credit period. For convenience, the demand rate *D*(*t*) may be given by
  1 $$D(M) = Ke^{aM} ,$$
  where, $$K > 0$$, $$a{ \ge }0$$.

The basis of the simple demand expression depends on two assumptions. One is the impact of trade credit on the demand, the other is the uncertainty of environment influence on the expectation value of the demand is zero (Jeuland and Shugan, 1983).

(ii) The longer the trade credit period is to the retailer, the higher the default risk is to the supplier. The default risk function with respect to trade credit period is given by
  2 $$F(M) = 1 - e^{ - bM} ,$$
  where, $$b{ \ge }0$$.
(iii) Shortages are not permitted and lead time is zero.
(v) The replenishment is instantaneous and the production rate is finite. Furthermore, the demand for the product does not exceed the production rate in model.
(vi) The supplier follows a lot-for-lot replenishment policy.
(vii) To simplify the problem and obtain main conclusions, we further assume that the retailer’s capital opportunity cost equal to its opportunity gain.

### Notation

For convenience, subscript *i* represents different member, $i = s$ represents the supplier; $i = r$ represents the retailer; $i = sc$ represents the whole supply chain.

*A*: the production rate per year for the supplier.
*b*: the default risk coefficient.
*Ke*^*aM*^: the demand rate per year, $$A \ge Ke^{aM}$$, $$M_{\rm max} = {{\ln \left( {{A \mathord{\left/ {\vphantom {A K}} \right. \kern-0pt} K}} \right)} \mathord{\left/ {\vphantom {{\ln \left( {{A \mathord{\left/ {\vphantom {A K}} \right. \kern-0pt} K}} \right)} a}} \right. \kern-0pt} a}$$, where, $$K$$ the basic demand rate, $$a$$ the increasing demand coefficient.
*S*_*i*_: the setup cost, $/order, $$i = s,r$$.
*C*: the production cost per unit, $/unit.
*W*: the wholesale price per unit, $/unit.
*P*: the retail price per unit, $/unit, with $$P > W > C$$.
*h*_*i*_: the inventory holding cost, $/unit/year, $$i = s,r$$.
*I*_*i*_: the interest charged per dollar per year, $/year, $$i = s,r$$.
$$\Pi_{i}^{j}$$
total annual profit. $$i = s,r,sc$$, $$j = 0$$, 1, 2, 3. $$j = 0$$ decentralized decision; $$j = 1$$ centralized decision; $$j = 2$$ the Nash game; $$j = 3$$ the supplier-Stackelberg game.
$$M^{j}$$
the length of the trade credit period offered by the supplier in years, decision variable, $$j = 2$$, 3.
$$Q^{j}$$
the order quantity, decision variable, $$j = 0$$, 1, 2, 3.

## Mathematical formulation of the model without trade credit

In this section, we first propose two inventory models without trade credit, i.e., decentralized decision and centralized decision. The corresponding results of the two scenarios will be used as comparison benchmarks when the supplier permits delay in payments to the retailer for supply chain coordination.

Firstly, in the decentralized decision, there is no coordination and no trade credit between the supplier and the retailer. Therefore, the demand rate is constant $K$. The retailer adopts the classic EOQ solution to optimize its total profit or total cost. The optimal economic order quantity (EOQ) of the retailer is given by

3 $$Q^{0*} = \sqrt {2KS_{r} /h_{r} } ,$$

and the optimal annual profit of the retailer is

4 $$\Pi_{r}^{0} = \left( {P - W} \right)K - \sqrt {2KS_{r} h_{r} } .$$

When the retailer orders $Q^{0*}$, the supplier will produce lot size $Q^{0*}$ and instantaneously replenishes the retailer according to the lot-for-lot policy. Therefore, the supplier’s total annual profit is expressed as

5 $$\Pi_{s}^{0} = \left( {W - C} \right)K - S_{s} \sqrt {Kh_{r} /2S_{r} } - Kh_{s} \sqrt {KS_{r} /2h_{r} } /A.$$

Consequently, the total annual profit of the supply chain is given by

6 $$\Pi_{sc}^{0} = \left( {P - C} \right)K - \sqrt {2KS_{r} h_{r} } - S_{s} \sqrt {Kh_{r} /2S_{r} } - Kh_{s} \sqrt {KS_{r} /2h_{r} } /A.$$

Secondly, in the centralized decision, the supplier and the retailer are willing to collaborate and form a vertical alliance or virtual integrated firm. They will jointly decide the replenishment schedule. For no trade credit, the demand rate is still constant, $K$. In this way, the joint total annual profit for the whole supply chain is

7 $$\Pi_{sc}^{1} (Q) = \left( {P - C} \right)K - K\left( {S_{r} + S_{s} } \right)/Q - Qh_{r} /2 - KQh_{s} /2A.$$

Therefore, the optimal joint order quantity of the supply chain is given by

8 $$Q^{1*} = \sqrt {2AK\left( {S_{r} + S_{s} } \right)/\left( {Ah_{r} + Kh_{s} } \right)} .$$

The optimal annual profit of the supply chain responding to lot size $Q^{1*}$ will be

9 $$\Pi_{sc}^{1} = \left( {P - C} \right)K - \sqrt {2K\left( {S_{r} + S_{s} } \right)\left( {Ah_{r} + Kh_{s} } \right)/A} .$$

## Mathematical formulation of the models with trade credit

According to the assumptions (i) and (ii), the supplier’s expected net revenue with default risk is $WD(M)\left( {1 - F(M)} \right) = WKe^{{\left( {a - b} \right)M}}$. Additionally, he or she will burden an additional capital opportunity cost, i.e., $CKe^{aM} I_{s} M$, for offering trade credit period $M$ to the retailer. Meanwhile, for the retailer, he or she can save an additional capital opportunity cost $WKe^{aM} I_{r} M$. Therefore, the retailer’s and the supplier’s total annual profits can be expressed as

10 $$\Pi_{r}^{2} (Q) = \Pi_{r}^{3} (Q) = \left( {P - W} \right)Ke^{aM} - S_{r} Ke^{aM} /Q - Qh_{r} /2 + WKe^{aM} I_{r} M,$$

11 $$\Pi_{s}^{2} (M) = \Pi_{s}^{3} (M) = WKe^{{\left( {a - b} \right)M}} - CKe^{aM} - \frac{{Ke^{aM} S_{s} }}{Q} - \frac{{Ke^{aM} h_{s} Q}}{2A} - CKe^{aM} I_{s} M,$$

respectively.

### Two parties’ decision making in Nash game

In this subsection, we assume that the supplier and the retailer have the same bargaining power, i.e., neither side has the monopoly strength. Under this background, the optimal equilibrium solution is Nash equilibrium.

From the view of Nash game, the first derivative condition of $\Pi_{r}^{2} (Q)$ with respect to $Q$ and the first derivative condition of $\Pi_{s}^{2} (M)$ with respect to $M$ should be established simultaneously. Therefore, the first derivative ${{d\Pi_{r}^{2} (Q)} \mathord{\left/ {\vphantom {{d\Pi_{r}^{2} (Q)} {dQ}}} \right. \kern-0pt} {dQ}}$ and the first derivative $d\Pi_{s}^{2} (M)/dM$ will be given by

12 $$d\Pi_{r}^{2} (Q)/dQ = S_{r} Ke^{aM} /Q^{2} - h_{r} /2,$$

13 $$\frac{{d\Pi_{s}^{2} (M)}}{dM} = \left( {a - b} \right)WKe^{{\left( {a - b} \right)M}} - aCKe^{aM} - \frac{{aKe^{aM} S_{s} }}{Q} - \frac{{aKe^{aM} h_{s} Q}}{2A} - CKe^{aM} I_{s} - aCKe^{aM} I_{s} M.$$

First, by the first derivative condition $d\Pi_{r}^{2} (Q)/dQ = 0$, the optimal ordering lot size in Nash game is given by

14 $$Q^{2*} = \sqrt {2S_{r} Ke^{aM} /h_{r} } .$$

Next, substituting $Q^{2*} = \sqrt {2S_{r} Ke^{aM} /h_{r} }$ into Eq. (13), the $d\Pi_{s}^{2} (M)/dM$ may be reduced to

15 $$\frac{{d\Pi_{s}^{2} (M)}}{dM} = \left( {a - b} \right)WKe^{{\left( {a - b} \right)M}} - aCKe^{aM} - aS_{s} \sqrt {\frac{{h_{r} Ke^{aM} }}{{2S_{r} }}} - \frac{{aKe^{aM} h_{s} }}{A}\sqrt {\frac{{S_{r} Ke^{aM} }}{{2h_{r} }}} - CKe^{aM} I_{s} - aCKe^{aM} I_{s} M.$$

It includes a single decision variable $M$.

#### **Theorem 1**

*The supplier’s optimal trade credit period is zero (i.e.,* $M^{2*} = 0$ *) if (i)* $a \le b$ *, or (ii)* $(a - b)W \le aC$ *, or (iii)* $(a - b)W \le aC + CI_{s}$.

#### *Proof*

From Eq. (15), if $a \le b$, $\frac{{d\Pi_{s}^{2} (M)}}{dM} < 0$. Consequently, the optimal trade credit period is zero, i.e., $M^{2*} = 0$. Similarly, if $(a - b)W \le aC$, or $(a - b)W \le aC + CI_{s}$, we have the same results $\frac{{d\Pi_{s}^{2} (M)}}{dM} < 0$, and $M^{2*} = 0$. This completes the proof.

Consequently, the retailer’s and the supplier’s total annual profits are given by

16 $$\Pi_{r}^{2} (\text{M}^{{\text{2*}}} = 0) = \left( {P - W} \right)K - \sqrt {2KS_{r} h_{r} } = \Pi_{r}^{0} ,$$

17 $$\Pi_{s}^{2} (\text{M}^{{\text{2*}}} = 0) = \left( {W - C} \right)K - S_{s} \sqrt {{{Kh_{r} } \mathord{\left/ {\vphantom {{Kh_{r} } {2S_{r} }}} \right. \kern-0pt} {2S_{r} }}} - {{Kh_{s} \sqrt {{{KS_{r} } \mathord{\left/ {\vphantom {{KS_{r} } {2h_{r} }}} \right. \kern-0pt} {2h_{r} }}} } \mathord{\left/ {\vphantom {{Kh_{s} \sqrt {{{KS_{r} } \mathord{\left/ {\vphantom {{KS_{r} } {2h_{r} }}} \right. \kern-0pt} {2h_{r} }}} } A}} \right. \kern-0pt} A} = \Pi_{s}^{0} .$$

That is to say, the two parties don’t achieve any coordination or improvement in Theorem 1.

Next, we discuss the another condition, i.e., $(a - b)W > aC + CI_{s}$. By the first derivative condition ${{d\Pi_{s}^{2} (M)} \mathord{\left/ {\vphantom {{d\Pi_{s}^{2} (M)} {dM = 0}}} \right. \kern-0pt} {dM = 0}}$, we can obtain

18 $$\left( {a - b} \right)We^{ - bM} - aC - aS_{s} \sqrt {\frac{{h_{r} }}{{2S_{r} Ke^{aM} }}} - \frac{{ah_{s} }}{A}\sqrt {\frac{{S_{r} Ke^{aM} }}{{2h_{r} }}} - CI_{s} - aCI_{s} M = 0.$$

From Eq. (18), the optimal trade credit period function is given by

19 $$M^{{2\bar{*}}} = {{\left\{ {\left( {a - b} \right)We^{{ - bM^{{2\bar{*}}} }} - aC - aS_{s} \sqrt {\frac{{h_{r} }}{{2S_{r} Ke^{{aM^{{2\bar{*}}} }} }}} - \frac{{ah_{s} }}{A}\sqrt {\frac{{S_{r} Ke^{{aM^{{2\bar{*}}} }} }}{{2h_{r} }}} - CI_{s} } \right\}} \mathord{\left/ {\vphantom {{\left\{ {\left( {a - b} \right)We^{{ - bM^{{2\bar{*}}} }} - aC - aS_{s} \sqrt {\frac{{h_{r} }}{{2S_{r} Ke^{{aM^{{2\bar{*}}} }} }}} - \frac{{ah_{s} }}{A}\sqrt {\frac{{S_{r} Ke^{{aM^{{2\bar{*}}} }} }}{{2h_{r} }}} - CI_{s} } \right\}} {aCI_{s} }}} \right. \kern-0pt} {aCI_{s} }},$$

$${\text{when}}\;\left( {a - b} \right)We^{{ - bM^{{2\bar{*}}} }} - aC - aS_{s} \sqrt {\frac{{h_{r} }}{{2S_{r} Ke^{{aM^{{2\bar{*}}} }} }}} - \frac{{ah_{s} }}{A}\sqrt {\frac{{S_{r} Ke^{{aM^{{2\bar{*}}} }} }}{{2h_{r} }}} - CI_{s} > 0.$$

Note that the left and right sides of Eq. (19) are functions of $M$. Due to the complexity of the problem, it seems difficult to derive a closed-form expression. Additionally, in Eq. (19), we obviously observe that the left side increases and the right side decreases as $M$ increases. There is only one intersection point when the two sides of Eq. (19) intersect, i.e., unique optimal positive solution $M^{{2\bar{*}}}$.

#### **Theorem 2**

*When* $\left( {a - b} \right)W - aC - CI_{s} - aS_{s} \sqrt {\frac{{h_{r} }}{{2S_{r} K}}} - \frac{{ah_{s} }}{A}\sqrt {\frac{{S_{r} K}}{{2h_{r} }}} > 0$ *, (i) if* $M^{{2\bar{*}}} < M_{\rm max}$ *, the final optimal trade credit period is* $M^{2*} = M^{{2\bar{*}}}$ *; (ii) if* $M^{{2\bar{*}}} \ge M_{\rm max}$ *, the final optimal trade credit period is* $M^{2*} = M_{\rm max}$.

#### *Proof*

Firstly, according to $\left( {a - b} \right)W - aC - CI_{s} - aS_{s} \sqrt {\frac{{h_{r} }}{{2S_{r} K}}} - \frac{{ah_{s} }}{A}\sqrt {\frac{{S_{r} K}}{{2h_{r} }}} > 0$, we can obtain that

20 $$\frac{{d\Pi_{s}^{2} (M)}}{dM}\left| {_{M = 0}^{{}} } \right. = (a - b)WK - aCK - aS_{s} \sqrt {\frac{{h_{r} K}}{{2S_{r} }}} - \frac{{aKh_{s} }}{A}\sqrt {\frac{{S_{r} K}}{{2h_{r} }}} - CKI_{s} > 0,$$

21 $$\frac{{d\Pi_{s}^{2} (M)}}{dM}\left| {_{M \to \infty }^{{}} } \right. = - \infty .$$

Additionally, applying the second derivative of $\Pi_{s}^{2} (M)$ with respect to $M$, we have

22 $$\frac{{d^{2} \Pi_{s}^{2} (M)}}{{dM^{2} }} = \left[ {(a - b)^{2} We^{ - bM} - a^{2} C - \frac{{3a^{2} h_{s} }}{2A}\sqrt {\frac{{S_{r} Ke^{aM} }}{{2h_{r} }}} - 2aCI_{s} } \right]Ke^{aM} - a^{2} S_{s} \sqrt {\frac{{h_{r} Ke^{aM} }}{{8S_{r} }}} - a^{2} CKe^{aM} I_{s} M.$$

Next, we have two alternative cases: (i) $(a - b)^{2} W - a^{2} C - \frac{{3a^{2} h_{s} }}{2A}\sqrt {\frac{{S_{r} K}}{{2h_{r} }}} - 2aCI_{s} \le 0$ and (ii) $(a - b)^{2} W - a^{2} C - \frac{{3a^{2} h_{s} }}{2A}\sqrt {\frac{{S_{r} K}}{{2h_{r} }}} - 2aCI_{s} > 0$.

#### Case 1

$$(a - b)^{2} W - a^{2} C - \frac{{3a^{2} h_{s} }}{2A}\sqrt {\frac{{S_{r} K}}{{2h_{r} }}} - 2aCI_{s} \le 0$$.

In this case, we know that $(a - b)^{2} We^{ - bM} - a^{2} C - \frac{{3a^{2} h_{s} }}{2A}\sqrt {\frac{{S_{r} Ke^{aM} }}{{2h_{r} }}} - 2aCI_{s} < 0$, further, $\frac{{d^{2} \Pi_{s}^{2} (M)}}{{dM^{2} }} < 0$. Therefore, $\Pi_{s}^{2} (M)$ is a strictly concave function in $\left[ {0,\infty } \right)$. Therefore, combining with Eq. (20) and Eq. (21), we know that there exists a unique positive optimal solution such that $\frac{{d\Pi_{s}^{2} (M)}}{dM} = 0$, denoted as $M^{{2\bar{*}}}$.

#### Case 2

$$(a - b)^{2} W - a^{2} C - \frac{{3a^{2} h_{s} }}{2A}\sqrt {\frac{{S_{r} K}}{{2h_{r} }}} - 2aCI_{s} > 0$$

In this case, we know that the value of $\frac{{d^{2} \Pi_{s}^{2} (M)}}{{dM^{2} }}$ moves from positive to negative as $M$ increases, that is to say, $\Pi_{s}^{2} (M)$ is a convex-concave function of $M$. Therefore, combining with Eq. (20) and Eq. (21), we know that $\Pi_{s}^{2} (M)$ is a unimodal function in $\left[ {0,\infty } \right)$. There also exists a unique positive optimal solution such that $\frac{{d\Pi_{s}^{2} (M)}}{dM} = 0$, denoted as $M^{{2\bar{*}}}$.

In a word, if $\left( {a - b} \right)W - aC - CI_{s} - aS_{s} \sqrt {\frac{{h_{r} }}{{2S_{r} K}}} - \frac{{ah_{s} }}{A}\sqrt {\frac{{S_{r} K}}{{2h_{r} }}} > 0$, the solution of Eq. (19) is a unique optimal positive solution $M^{{2\bar{*}}}$ for $\Pi_{s}^{2} (M)$. Then, we consider the upper bound of $M$, i.e., $M_{\rm max}$. If $M^{{2\bar{*}}} < M_{\rm max}$, the final optimal trade credit period is $M^{2*} = M^{{2\bar{*}}}$. If $M^{{2\bar{*}}} \ge M_{\rm max}$, the final optimal trade credit period is $M^{2*} = M_{\rm max}$. This completes the proof.

From Eq. (19) and Theorem 2, we obtain the following results.

#### **Corollary 1**

*(i) A higher value of* $a$, $W$, $A$ *and a lower value of* $b$, $C$, $S_{s}$, $h_{s}$, $I_{s}$ *cause a higher value of* $\left( {a - b} \right)W - aC - CI_{s} - aS_{s} \sqrt {\frac{{h_{r} }}{{2S_{r} K}}} - \frac{{ah_{s} }}{A}\sqrt {\frac{{S_{r} K}}{{2h_{r} }}}$, *and* $M^{{2\bar{*}}}$.

*(ii) The change of* $P$ *and* $I_{r}$, *i.e., the retailer’s profit parameters, do not affect the supplier as to whether to offer trade credit to the retailer.*

#### *Proof*

The above is apparent from $\left( {a - b} \right)W - aC - CI_{s} - aS_{s} \sqrt {\frac{{h_{r} }}{{2S_{r} K}}} - \frac{{ah_{s} }}{A}\sqrt {\frac{{S_{r} K}}{{2h_{r} }}} > 0$, Eq. (19) and Theorem 2.

A simple economic interpretation is as follows. A higher value of $a$ (i.e., increasing demand coefficient) leads to a higher demand, and higher values of $W$ and $A$ lead to higher revenue. Hence, the supplier is willing to offer a longer trade credit period. On the other hand, lower values of $b$ (i.e., default risk coefficient) and $C$ lead to a higher expected revenue for supplier, and lower values of $S_{s}$, $h_{s}$, and $I_{s}$ lead to a lower ordering and inventory cost. Hence, the supplier willing to offer a longer trade credit period to the retailer.

Furthermore, according to Theorem 2, Theorem 1 can be modified to Theorem 3.

#### **Theorem 3**

*The supplier’s optimal trade credit period is zero (i.e.,* $M^{2*} = 0$ *) if (i)* $a \le b$ *, or (ii)* $(a - b)W \le aC$ *, or (iii)* $(a - b)W \le aC + CI_{s}$ *, or (iv)* $\left( {a - b} \right)W - aC - CI_{s} - aS_{s} \sqrt {\frac{{h_{r} }}{{2S_{r} K}}} - \frac{{ah_{s} }}{A}\sqrt {\frac{{S_{r} K}}{{2h_{r} }}} \le 0$.

#### **Corollary 2**

*The supplier’s optimal trade credit period is zero (i.e.,* $M^{2*} = 0$ *) if* $\left( {a - b} \right)W - aC - CI_{s} - a\sqrt {\frac{{S_{s} h_{s} }}{A}} \le 0$.

#### *Proof*

We use the theorem that the arithmetic mean is not always less than the geometric mean. It is omitted.

In a word, the retailer’s and the supplier’s final total annual profits in Nash game are given by

23 $$\Pi_{r}^{2} (M^{2*} ) = \left( {P - W} \right)Ke^{{aM^{2*} }} - \sqrt {2S_{r} h_{r} Ke^{{aM^{2*} }} } + WKe^{{aM^{2*} }} I_{r} M^{2*} ,$$

24 $$\Pi_{s}^{2} (M^{2*} ) = WKe^{{\left( {a - b} \right)M^{2*} }} - CKe^{{aM^{2*} }} - S_{s} \sqrt {\frac{{Ke^{{aM^{2*} }} h_{r} }}{{2S_{r} }}} - \frac{{Ke^{{aM^{2*} }} h_{s} }}{A}\sqrt {\frac{{Ke^{{aM^{2*} }} S_{r} }}{{2h_{r} }}} - CKe^{{aM^{2*} }} I_{s} M^{2*} ,$$

respectively.

Note that $\Pi_{r}^{2} (M^{2*} )$ is an increasing function of $M^{2*}$ only if $\Pi_{r}^{0} \ge 0$, which is a reasonable assumption. That is to say, as long as the trade credit period is offered by the supplier, $\Pi_{r}^{2} (M^{2*} )$ is greater than $\Pi_{r}^{0}$, i.e., $\Pi_{r}^{2} (M^{2*} ) \ge \Pi_{r}^{0}$. Additionally, it is obvious that $\Pi_{s}^{2} (M^{2*} ) \ge \Pi_{s}^{0}$. Proof is omitted.

### Two parties’ decision making in a supplier-Stackelberg game

In this subsection, we suppose that the supplier is the dominating company over the retailer. For example, a supplier, such as Siwin Foods, (a famous food manufacturer in China) has a dominate power over its downstream small store. Consequently, the dominating company (e.g., Siwin Foods) acts as a leader, its downstream small store acts as a follower, which call a supplier-Stackelberg game. In a supplier-Stackelberg game, the supplier offers a trade credit period $M$, and then the retailer maximizes his or her own profit to find optimal ordering lot size, next, the supplier observes the retailer’s optimal solution as a function of $M$, finally, he or she find the optimal $M$.

- *(i) The retailer’s optimal response*

Firstly, we should know how the retailer responds to any trade credit period $M$ offered by the supplier. By the first derivative necessary condition ${{d\Pi_{r}^{3} (Q)} \mathord{\left/ {\vphantom {{d\Pi_{r}^{3} (Q)} {dQ = 0}}} \right. \kern-0pt} {dQ = 0}}$, the optimal ordering lot size in a supplier-Stackelberg game is given by

25 $$Q^{3*} = \sqrt {{{2S_{r} Ke^{aM} } \mathord{\left/ {\vphantom {{2S_{r} Ke^{aM} } {h_{r} }}} \right. \kern-0pt} {h_{r} }}},$$

which is a function of $M$.

- *(ii) The supplier’s optimization*

After observing the optimal response of the retailer (given by Eq. (25)), the supplier selects optimal $M$ so that his or her total annual profit is maximized.

Therefore, substituting $Q^{3*} = \sqrt {{{2S_{r} Ke^{aM} } \mathord{\left/ {\vphantom {{2S_{r} Ke^{aM} } {h_{r} }}} \right. \kern-0pt} {h_{r} }}}$ into Eq. (11), the $\Pi_{s}^{3} (M)$ can be modified to a new function of $M$ will be given by

26 $$\Pi_{s}^{3} (M) = WKe^{{\left( {a - b} \right)M}} - CKe^{aM} - S_{s} \sqrt {\frac{{h_{r} Ke^{aM} }}{{2S_{r} }}} - \frac{{Ke^{aM} h_{s} }}{2A}\sqrt {\frac{{2Ke^{aM} S_{r} }}{{h_{r} }}} - CKe^{aM} I_{s} M.$$

In order to maximize $\Pi_{s}^{3} (M)$ in Eq. (26), we obtain

27 $$\frac{{d\Pi_{s}^{3} (M)}}{dM} = \left( {a - b} \right)WKe^{{\left( {a - b} \right)M}} - aCKe^{aM} - \frac{{aS_{s} }}{2}\sqrt {\frac{{h_{r} Ke^{aM} }}{{2S_{r} }}} - \frac{{3ah_{s} Ke^{aM} }}{2A}\sqrt {\frac{{S_{r} Ke^{aM} }}{{2h_{r} }}} - CKe^{aM} I_{s} - aCKe^{aM} I_{s} M$$

#### **Theorem 4**

*The supplier’s optimal trade credit period is zero (i.e.,* $M^{3*} = 0$ *) if (i)* $a \le b$ *, or (ii)* $(a - b)W \le aC$ *, or (iii)* $(a - b)W \le aC + CI_{s}$.

#### *Proof*

We omit the proof of Theorem 4 since it mimics that of Theorem 1.

Consequently, the retailer’s and the supplier’s total annual profits are given by

28 $$\Pi_{r}^{3} (M^{3*} = 0) = \left( {P - W} \right)K - \sqrt {2KS_{r} h_{r} } = \Pi_{r}^{0} ,$$

29 $$\Pi_{s}^{3} (M^{3*} = 0) = \left( {W - C} \right)K - S_{s} \sqrt {{{Kh_{r} } \mathord{\left/ {\vphantom {{Kh_{r} } {2S_{r} }}} \right. \kern-0pt} {2S_{r} }}} - {{Kh_{s} \sqrt {{{KS_{r} } \mathord{\left/ {\vphantom {{KS_{r} } {2h_{r} }}} \right. \kern-0pt} {2h_{r} }}} } \mathord{\left/ {\vphantom {{Kh_{s} \sqrt {{{KS_{r} } \mathord{\left/ {\vphantom {{KS_{r} } {2h_{r} }}} \right. \kern-0pt} {2h_{r} }}} } A}} \right. \kern-0pt} A} = \Pi_{s}^{0} .$$

That is to say, the two parties don’t achieve any coordination or improvement in Theorem 4.

Next, we discuss the another condition, i.e., $(a - b)W > aC + CI_{s}$. By the first derivative condition ${{d\Pi_{s}^{3} (M)} \mathord{\left/ {\vphantom {{d\Pi_{s}^{3} (M)} {dM = 0}}} \right. \kern-0pt} {dM = 0}}$, we obtain

30 $$\left( {a - b} \right)We^{ - bM} - aC - \frac{{aS_{s} }}{2}\sqrt {\frac{{h_{r} }}{{2S_{r} Ke^{aM} }}} - \frac{{3ah_{s} }}{2A}\sqrt {\frac{{S_{r} Ke^{aM} }}{{2h_{r} }}} - CI_{s} - aCI_{s} M = 0.$$

From Eq. (30), the optimal trade credit period function is given by

31 $$M^{{3\bar{*}}} = {{\left\{ {\left( {a - b} \right)We^{{ - bM^{{3\bar{*}}} }} - aC - \frac{{aS_{s} }}{2}\sqrt {\frac{{h_{r} }}{{2S_{r} Ke^{{aM^{{3\bar{*}}} }} }}} - \frac{{3ah_{s} }}{2A}\sqrt {\frac{{S_{r} Ke^{{aM^{{3\bar{*}}} }} }}{{2h_{r} }}} - CI_{s} } \right\}} \mathord{\left/ {\vphantom {{\left\{ {\left( {a - b} \right)We^{{ - bM^{{3\bar{*}}} }} - aC - \frac{{aS_{s} }}{2}\sqrt {\frac{{h_{r} }}{{2S_{r} Ke^{{aM^{{3\bar{*}}} }} }}} - \frac{{3ah_{s} }}{2A}\sqrt {\frac{{S_{r} Ke^{{aM^{{3\bar{*}}} }} }}{{2h_{r} }}} - CI_{s} } \right\}} {aCI_{s} }}} \right. \kern-0pt} {aCI_{s} }},$$

$${\text{when}}\;\left( {a - b} \right)We^{{ - bM^{{3\bar{*}}} }} - aC - \frac{{aS_{s} }}{2}\sqrt {\frac{{h_{r} }}{{2S_{r} Ke^{{aM^{{3\bar{*}}} }} }}} - \frac{{3ah_{s} }}{2A}\sqrt {\frac{{S_{r} Ke^{{aM^{{3\bar{*}}} }} }}{{2h_{r} }}} - CI_{s} > 0.$$

#### **Theorem 5**

*When* $\left( {a - b} \right)W - aC - CI_{s} - \frac{{aS_{s} }}{2}\sqrt {\frac{{h_{r} }}{{2S_{r} K}}} - \frac{{3ah_{s} }}{2A}\sqrt {\frac{{S_{r} K}}{{2h_{r} }}} > 0$ *, (i) if* $M^{{3\bar{*}}} < M_{\rm max}$ *, the final optimal trade credit period is* $M^{3*} = M^{{3\bar{*}}}$ *; (ii) if* $M^{{3\bar{*}}} \ge M_{\rm max}$ *, the final optimal trade credit period is* $M^{3*} = M_{\rm max}$.

#### *Proof*

We omit the proof of Theorem 5 since it mimics that of Theorem 2.

From Eq. (31) and Theorem 5, we can obtain the following results.

#### **Corollary 3**

- *(i) A higher value of* $a$, $W$, $A$ *and a lower value of* $b$, $C$, $S_{s}$, $h_{s}$, $I_{s}$ *cause a higher value of* $\left( {a - b} \right)W - aC - CI_{s} - \frac{{aS_{s} }}{2}\sqrt {\frac{{h_{r} }}{{2S_{r} K}}} - \frac{{3ah_{s} }}{2A}\sqrt {\frac{{S_{r} K}}{{2h_{r} }}}$ *and* $M^{{3\bar{*}}}$.
- *(ii) The change of* $P$ *and* $I_{r}$, *i.e., the retailer’s profit parameters, do not affect the supplier as to whether to offer trade credit to the retailer.*

#### *Proof*

It is omitted.

Likewise, according to Theorem 5, Theorem 4 can be modified to Theorem 6.

#### **Theorem 6**

*The supplier’s optimal trade credit period is zero (i.e.,* $\Pi_{r}^{3} (M^{3*} ) \ge \Pi_{r}^{0}$ *) if (i)* $a \le b$ *, or (ii)* $(a - b)W \le aC$ *, or (iii)* $(a - b)W \le aC + CI_{s}$ *, or (iv)* $\left( {a - b} \right)W - aC - CI_{s} - \frac{{aS_{s} }}{2}\sqrt {\frac{{h_{r} }}{{2S_{r} K}}} - \frac{{3ah_{s} }}{2A}\sqrt {\frac{{S_{r} K}}{{2h_{r} }}} \le 0$.

#### **Corollary 4**

*The supplier’s optimal trade credit period is zero (i.e.,* $M^{3*} = 0$ *) if* $\left( {a - b} \right)W - aC - CI_{s} - a\sqrt {\frac{{3S_{s} h_{s} }}{4A}} \le 0$.

#### *Proof*

It is omitted.

Consequently, the retailer’s and the supplier’s final total annual profits in a supplier-Stackelberg game are given by

32 $$\Pi_{r}^{3} (M^{3*} ) = \left( {P - W} \right)Ke^{{aM^{3*} }} - \sqrt {2S_{r} h_{r} Ke^{{aM^{3*} }} } + WKe^{{aM^{3*} }} I_{r} M^{3*} ,$$

33 $$\Pi_{s}^{3} (M^{3*} ) = WKe^{{\left( {a - b} \right)M^{3*} }} - CKe^{{aM^{3*} }} - S_{s} \sqrt {\frac{{Ke^{{aM^{3*} }} h_{r} }}{{2S_{r} }}} - \frac{{Ke^{{aM^{3*} }} h_{s} }}{A}\sqrt {\frac{{Ke^{{aM^{3*} }} S_{r} }}{{2h_{r} }}} - CKe^{{aM^{3*} }} I_{s} M^{3*} ,$$

respectively.

Likewize, $\Pi_{r}^{3} (M^{3*} )$ is an increasing function of $M^{3*}$ only if $\Pi_{r}^{0} \ge 0$. Therefore, as long as the trade credit period is offered by the supplier, $\Pi_{r}^{3} (M^{3*} )$ is greater than $\Pi_{r}^{0}$, i.e., $\Pi_{r}^{3} (M^{3*} ) \ge \Pi_{r}^{0}$. Additionally, it is obvious that $\Pi_{s}^{3} (M^{3*} ) \ge \Pi_{s}^{0}$. Proof is omitted.

## Numerical examples and analysis

According to the analysis and arguments in Sect. 4, when the final optimal trade credit period is zero, the retailer’s and the supplier’s total annual profits will not be improved. Therefore, the following numerical example is proposed to illustrate the improvement process for the two games.

### *Example*

Given $$A = 10000$$ units/year, $$K = 3600$$ units/year, $$a = 1$$, $$b = 0.3$$, $$P = \$ 35$$/unit, $$W = \$ 23$$/unit, $$C = \$ 12$$/unit, $$S_{r}$$ = $200/order, $$h_{r} = \$ 5$$/unit/year, $$I_{r} = 0.12$$/year, $$S_{s}$$ = $300/setup, $$h_{s} = \$ 4.5$$/unit/year, $$I_{s} = 0.1$$/year, respectively.

By applying the corresponding expressions, the results are obtained as follow.

In the decentralized decision, the economic order quantity $Q^{0*} = 537$ units, $\Pi_{r}^{0} = \$ 40517$/year, $\Pi_{s}^{0} = \$ 37153$/year, the annual profit of the supply chain $\Pi_{sc}^{0} = \$ 77670$/year.

In the centralized decision, the optimal joint order quantity $Q^{1*} = 737$ units. The optimal annual profit of the supply chain $\Pi_{sc}^{1} = \$ 77918$/year.

In Nash game, we obtain $\left( {a - b} \right)W - aC - CI_{s} - aS_{s} \sqrt {\frac{{h_{r} }}{{2S_{r} K}}} - \frac{{ah_{s} }}{A}\sqrt {\frac{{S_{r} K}}{{2h_{r} }}} = 2.22 > 0$. Consequently, $M^{{2\bar{*}}} = 0.3989$ years $= 145$ days, and $M_{\rm max} = 1.0217$ years. According to Theorem 2, for $M_{\rm max} > M^{{2\bar{*}}}$, the final optimal trade credit period $M^{2*} = M^{{2\bar{*}}} = 0.3989$ years. Consequently, $Q^{2*} = 655$ units; $D^{2} = 5365$ units/year, an increase of 49.03 % ($\frac{5365 - 3600}{3600}*100\,\% = 49.03\,\%$); $\Pi_{\text{r}}^{2} (M^{2*} ) = \$ 67010$/year, an increase of 65.39 % ($\frac{67010 - 40517}{40517}*100\,\% = 65.39\,\%$) from the decentralized decision; $\Pi_{\text{s}}^{2} (M^{2*} ) = \$ 39279$/year, an increase of 5.72 % ($\frac{39279 - 37153}{37153}*100\,\% = 5.72\,\%$) from the decentralized decision; $\Pi_{\text{sc}}^{2} (M^{2*} ) = \$ 106289$/year, an increase of 36.41 % ($\frac{106289 - 77918}{77918}*100\,\% = 36.41\,\%$) from the centralized decision. However, we notice that the default risk is ${\text{F}}^{ 2} = 11.3\,\%$.

In a supplier-Stackelberg game, we obtain $\left( {a - b} \right)W - aC - CI_{s} - \frac{{aS_{s} }}{2}\sqrt {\frac{{h_{r} }}{{2S_{r} K}}} - \frac{{3ah_{s} }}{2A}\sqrt {\frac{{S_{r} K}}{{2h_{r} }}} = 2.44 > 0$. Consequently, $M^{{3\bar{*}}} = 0.4273$ years $= 156$ days. According to Theorem 5, for $M_{\rm max} > M^{{3\bar{*}}}$, the final optimal trade credit period $M^{3*} = M^{{3\bar{*}}} = 0.4273$ years. Consequently, $Q^{3*} = 664$ units/order; $D^{3} = 5519$ units/year, an increase of 53.31 %; $\Pi_{\text{r}}^{3} (M^{3*} ) = \$ 69420$/year, an increase of 71.34 % from the decentralized decision; $\Pi_{\text{s}}^{3} (M^{3*} ) = \$ 39291$/year, an increase of 5.75 % from the decentralized decision; $\Pi_{\text{sc}}^{3} (M^{3*} ) = \$ 108711$/year, an increase of 39.97 % from the centralized decision. However, default risk is ${\text{F}}^{ 3} = 12\, \%$.

**Fig. 1:**
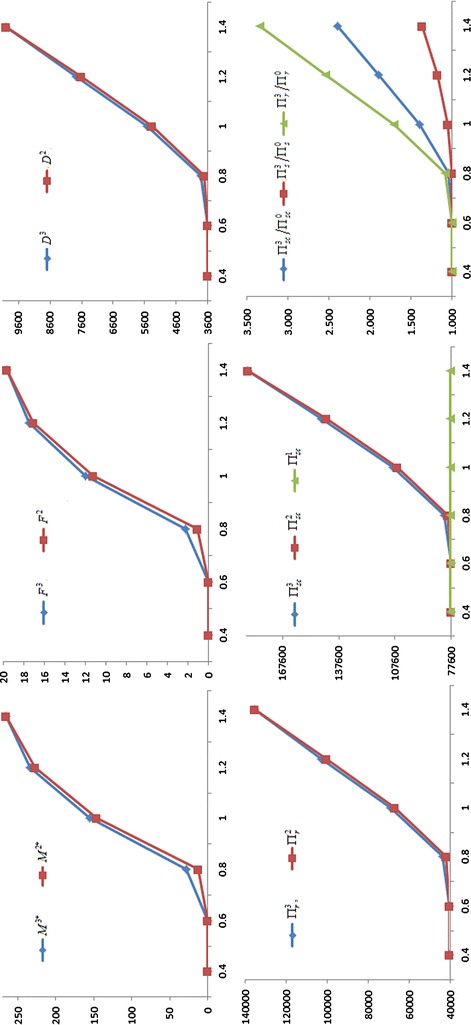
Influence of the increasing demand coefficient a on the optimal values

**Fig. 2:**
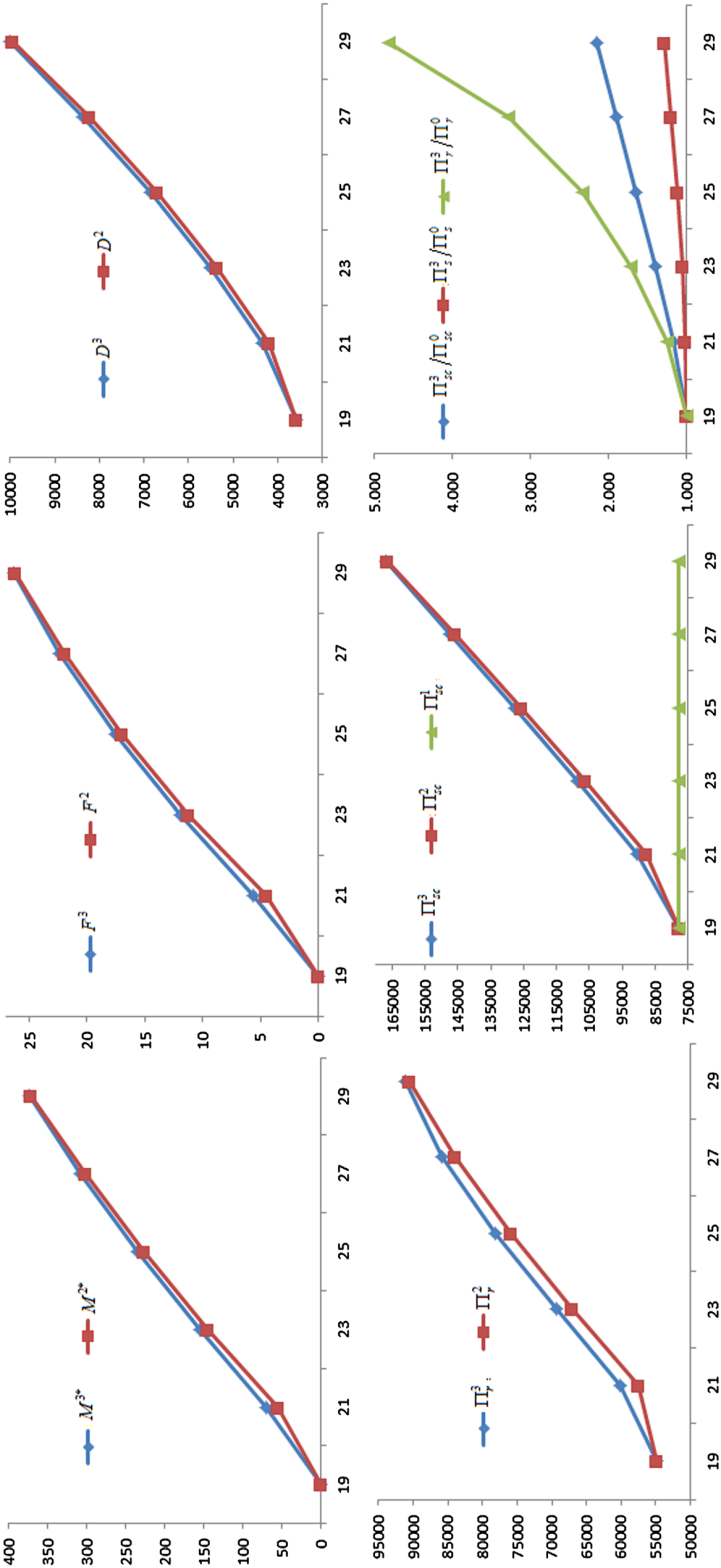
Influence of the wholesale price W on the optimal values

**Fig. 3:**
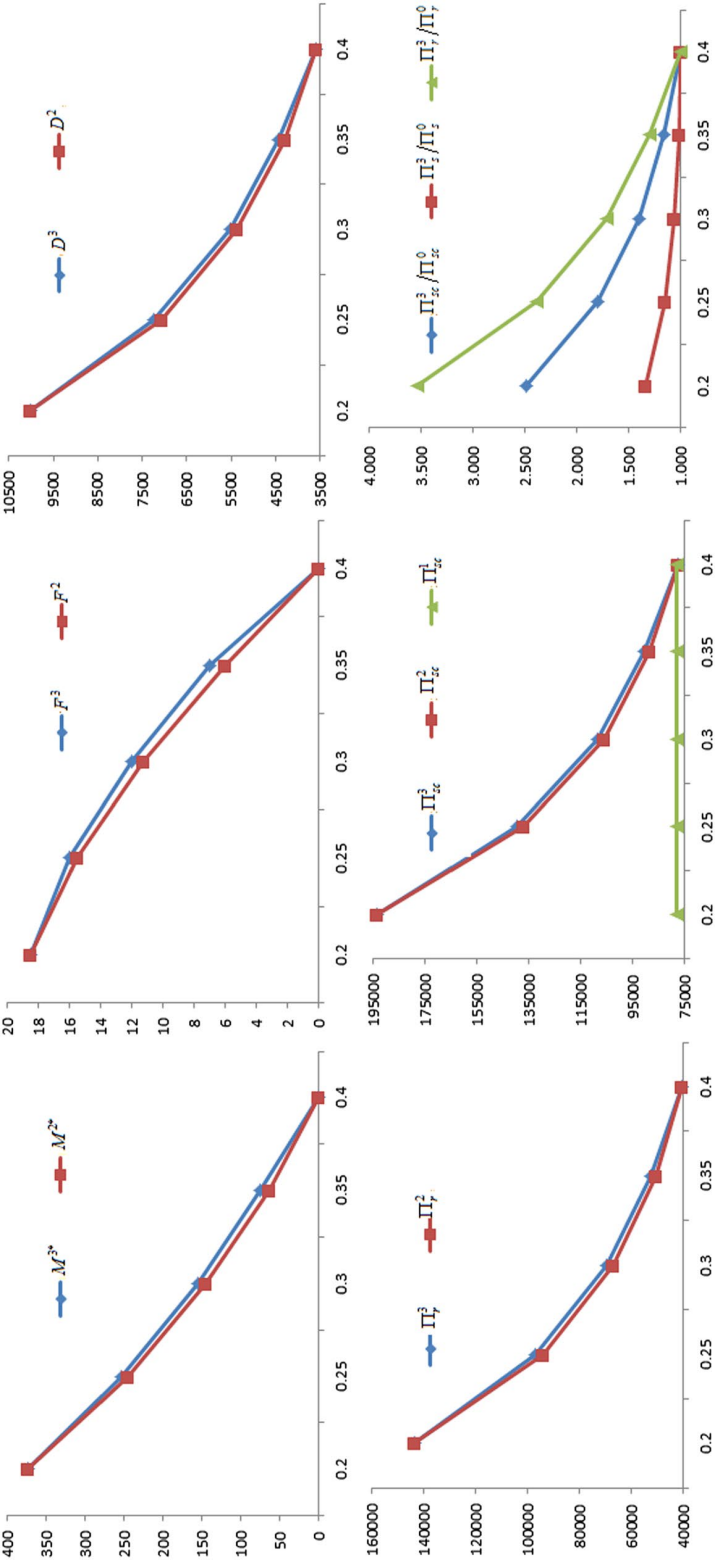
Influence of the default risk coefficient b on the optimal values

**Fig. 4:**
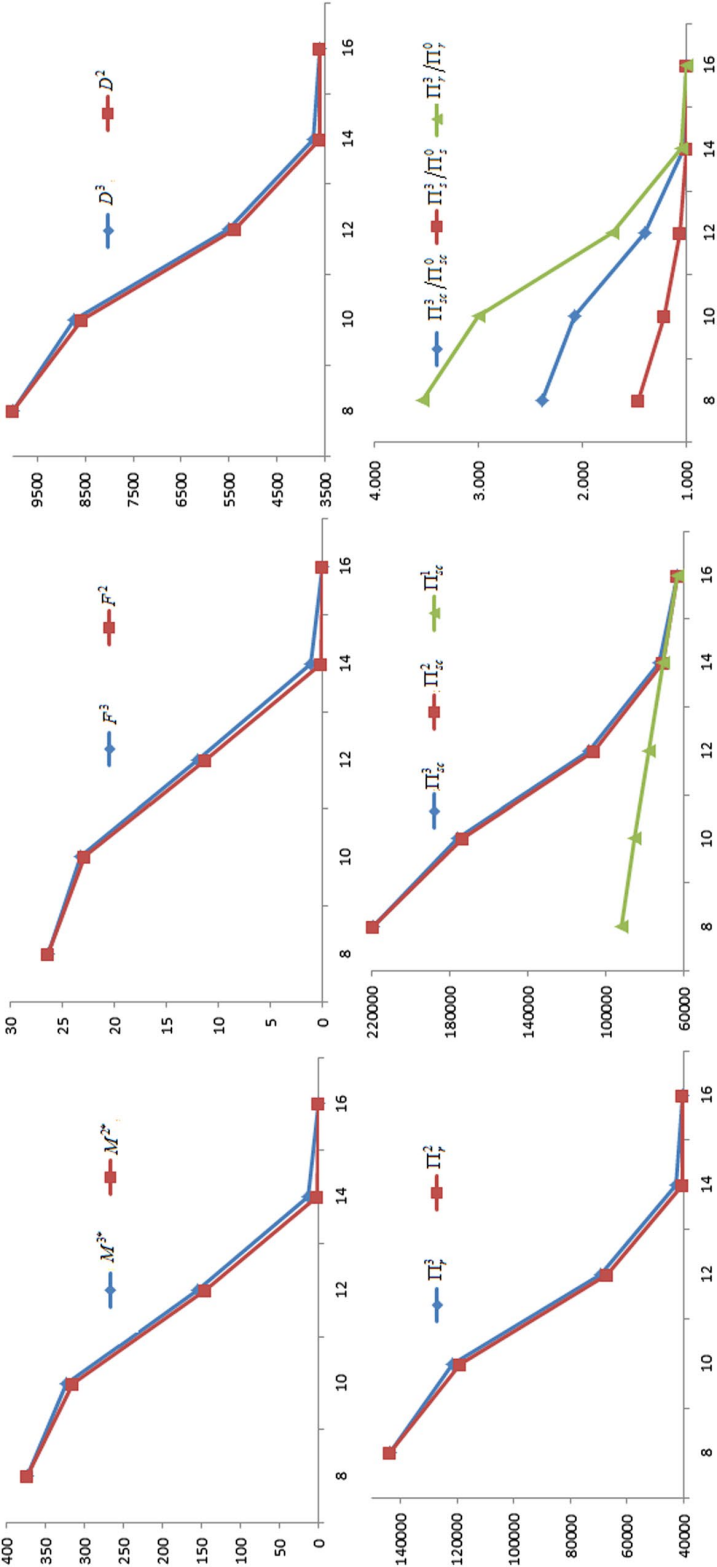
Influence of the production cost C on the optimal values

For gain more management insights, we present the sensitivity analysis to study the effects of changes in the values of parameters on the optimal values. The basic parameter values are the same as those in the Example. The computational results are given in Table 1. In addition, in order to better understanding, for *a*, *W*, *b* and $C$, the partial results of the sensitivity analysis have been graphed in Figs. 1, 2, 3 and 4 based on the data in Table 1. The rest of the results in Table 1 are not represented graphically, because the different is very small between the corresponding results of Nash game and supplier-Stackelberg game under the same conditions.

**Table 1:** The sensitivity analysis on parameters (Note that $M^{j*}$ is in days, $F^{j}$ is in percentage)

Parameters	Nash game						Decision without trade credit				Supplier-Stackelberg game						$\frac{{\Pi_{r}^{3} }}{{\Pi_{r}^{0} }}$	$\frac{{\Pi_{s}^{3} }}{{\Pi_{s}^{0} }}$	$\frac{{\Pi_{sc}^{3} }}{{\Pi_{sc}^{0} }}$
	$M^{2*}$	$F^{2}$	$D^{2}$	$\Pi_{r}^{2}$	$\Pi_{s}^{2}$	$\Pi_{sc}^{2}$	$\Pi_{r}^{0}$	$\Pi_{s}^{0}$	$\Pi_{sc}^{0}$	$\Pi_{sc}^{1}$	$M^{3*}$	$F^{3}$	$D^{3}$	$\Pi_{r}^{3}$	$\Pi_{s}^{3}$	$\Pi_{sc}^{3}$			
*a*																			
1.4	266	19.7	10,001	135,670	50,654	186,324	40,517	37,153	77,670	77,918	266	19.7	10,001	135,670	50,654	186,324	3.349	1.363	2.400
1.2	228	17.1	7606	100,451	43,880	144,331	40,517	37,153	77,670	77,918	234	17.5	7780	103,210	43,889	147,099	2.547	1.181	1.894
1	146	11.3	5365	67,010	39,279	106,289	40,517	37,153	77,670	77,918	156	12	5519	69,420	39,291	108,711	1.713	1.058	1.400
0.8	13	1.1	3707	42,141	37,187	79,328	40,517	37,153	77,670	77,918	28	2.3	3827	43,961	37,199	81,160	1.085	1.001	1.045
0.6	0	0	3600	40,517	37,153	77,670	40,517	37,153	77,670	77,918	0	0	3600	40,517	37,153	77,670	1.000	1.000	1.000
0.4	0	0	3600	40,517	37,153	77,670	40,517	37,153	77,670	77,918	0	0	3600	40,517	37,153	77,670	1.000	1.000	1.000
*A*																			
16,000	149	11.6	5421	67,885	39,588	107,473	40,517	37,316	77,833	78,148	162	12.4	5606	70,778	39,605	110,382	1.747	1.061	1.418
14,000	149	11.5	5408	67,674	39,514	107,188	40,517	37,277	77,794	78,092	160	12.3	5585	70,450	39,529	109,979	1.739	1.060	1.414
12,000	147	11.4	5390	67,395	39,416	106,811	40,517	37,225	77,742	78,019	158	12.2	5558	70,017	39,430	109,447	1.728	1.059	1.408
10,000	147	11.3	5365	67,010	39,279	106,289	40,517	37,153	77,670	77,918	156	12	5519	69,420	39,291	108,711	1.713	1.058	1.400
8000	143	11.1	5328	66,436	39,077	105,514	40,517	37,044	77,561	77,771	152	11.8	5463	68,543	39,087	107,629	1.692	1.055	1.388
6000	139	10.8	5268	65,503	38,747	104,250	40,517	36,863	77,380	77,535	146	11.3	5372	67,127	38,752	105,879	1.657	1.051	1.368
*W*																			
29	372	26.3	9962	90,595	75,818	166,412	18,917	58,753	77,670	77,918	373	26.4	10,000	91,081	75,820	166,901	4.815	1.291	2.149
27	302	22	8235	83,902	61,923	145,825	26,117	51,553	77,670	77,918	308	22.4	8372	85,782	61,930	147,711	3.285	1.201	1.902
25	227	17	6706	75,911	49,779	125,690	33,317	44,353	77,670	77,918	235	17.6	6855	78,092	49,788	127,881	2.344	1.123	1.647
23	146	11.3	5365	67,010	39,279	106,289	40,517	37,153	77,670	77,918	155	12	5519	69,420	39,291	108,711	1.713	1.058	1.400
21	56	4.5	4202	57,570	30,316	87,886	47,717	29,953	77,670	77,918	70	5.6	4357	60,137	30,331	90,468	1.260	1.013	1.165
19	0	0	3600	54,917	22,753	77,670	54,917	22,753	77,670	77,918	0	0	3600	54,917	22,753	77,670	1.000	1.000	1.000
*b*																			
0.4	0	0	3600	40,517	37,153	77,670	40,517	37,153	77,670	77,918	0	0	3600	40,517	37,153	77,670	1.000	1.000	1.000
0.35	64	6	4294	50,689	37,652	88,341	40,517	37,153	77,670	77,918	76	7	4431	52,732	37,665	90,397	1.302	1.014	1.164
0.3	146	11.3	5365	67,010	39,279	106,289	40,517	37,153	77,670	77,918	156	12	5519	69,420	39,291	108,711	1.713	1.058	1.400
0.25	246	15.5	7061	94,095	42,779	136,873	40,517	37,153	77,670	77,918	254	16	7230	96,865	42,788	139,653	2.391	1.152	1.798
0.2	373	18.5	10,000	143,730	49,868	193,598	40,517	37,153	77,670	77,918	373	18.5	10,000	143,730	49,868	193,598	3.547	1.342	2.493
*C*																			
16	0	0	3600	40,517	22,753	63,270	40,517	22,753	63,270	63,518	0	0	3600	40,517	22,753	63,270	1.000	1.000	1.000
14	1	0.1	3612	40,691	29,956	70,647	40,517	29,953	70,470	70,718	14	1.2	3743	42,579	29,970	72,549	1.051	1.001	1.030
12	146	11.3	5365	67,010	39,279	106,289	40,517	37,153	77,670	77,918	156	12	5519	69,420	39,291	108,711	1.713	1.058	1.400
10	316	22.9	8562	119,074	54,116	173,191	40,517	44,353	84,870	85,118	323	23.3	8722	121,782	54,123	175,905	3.006	1.220	2.073
8	373	26.4	10,000	143,730	75,745	219,475	40,517	51,553	92,070	92,318	373	26.4	10,000	143,730	75,745	219,475	3.547	1.469	2.384
*S*_*s*_																			
400	135	10.5	5212	64,632	38,436	103,069	40,517	36,482	76,999	77,452	151	11.7	5443	68,231	38,464	106,695	1.684	1.054	1.386
350	140	10.9	5288	65,817	38,858	104,675	40,517	36,817	77,334	77,680	153	11.8	5481	68,825	38,877	107,702	1.699	1.056	1.393
300	146	11.3	5365	67,010	39,279	106,289	40,517	37,153	77,670	77,918	156	12	5519	69,420	39,291	108,711	1.713	1.058	1.400
250	150	11.7	5442	68,207	39,701	107,908	40,517	37,488	78,005	78,169	158	12.2	5558	70,017	39,707	109,724	1.728	1.059	1.407
200	156	12	5519	69,412	40,122	109,534	40,517	37,824	78,340	78,434	161	12.4	5596	70,615	40,125	110,740	1.743	1.061	1.414
150	161	12.4	5596	70,624	40,543	111,167	40,517	38,159	78,676	78,716	163	12.6	5634	71,216	40,544	111,759	1.758	1.063	1.421
*h*_*s*_																			
5.5	143	11.1	5332	66,499	39,100	105,599	40,517	37,056	77,573	77,787	153	11.8	5469	68,639	39,109	107,748	1.694	1.055	1.389
5	144	11.2	5348	66,753	39,189	105,942	40,517	37,105	77,621	77,852	154	11.9	5494	69,027	39,200	108,227	1.704	1.057	1.394
4.5	146	11.3	5365	67,010	39,279	106,289	40,517	37,153	77,670	77,918	156	12	5519	69,420	39,291	108,711	1.713	1.058	1.400
4	147	11.4	5381	67,266	39,370	106,636	40,517	37,201	77,718	77,985	158	12.2	5545	69,817	39,383	109,200	1.723	1.059	1.405
3.5	148	11.4	5398	67,525	39,461	106,987	40,517	37,249	77,766	78,053	159	12.3	5570	70,218	39,476	109,694	1.733	1.060	1.411
3	149	11.5	5415	67,786	39,553	107,340	40,517	37,298	77,814	78,122	161	12.4	5596	70,624	39,569	110,194	1.743	1.061	1.416
*I*_*s*_																			
0.14	104	8.2	4785	58,091	38,356	96,447	40,517	37,153	77,670	77,918	114	9	4923	60,196	38,368	98,565	1.486	1.033	1.269
0.12	124	9.7	5053	62,183	38,769	100,952	40,517	37,153	77,670	77,918	134	10.4	5199	64,433	38,781	103,214	1.590	1.044	1.329
0.1	146	11.3	5365	67,010	39,279	106,289	40,517	37,153	77,670	77,918	156	12	5519	69,420	39,291	108,711	1.713	1.058	1.400
0.08	170	13	5733	72,778	39,910	112,688	40,517	37,153	77,670	77,918	180	13.8	5898	75,370	39,921	115,291	1.860	1.075	1.484
0.06	197	14.9	6176	79,790	40,688	120,478	40,517	37,153	77,670	77,918	207	15.7	6350	82,581	40,700	123,280	2.038	1.096	1.587
0.04	228	17.1	6715	88,476	41,655	130,131	40,517	37,153	77,670	77,918	237	17.7	6901	91,484	41,667	133,151	2.258	1.122	1.714

Underline is means the optimal trade credit period is $M_{\rm max}$

The sensitivity analysis reveals the following.

(i) $M^{2*}$, $F^{2}$, $D^{2}$, $\Pi_{r}^{2}$, $\Pi_{s}^{2}$, $M^{3*}$, $F^{3}$, $D^{3}$, $\Pi_{r}^{3}$, $\Pi_{s}^{3}$ increase as *a*, $A$ and $W$ increase, and decrease as $b$, $C$, $S_{s}$, $h_{s}$ and $I_{s}$ increase. This coincides with the Corollary 1 and Corollary3, and the purpose of two games. Note that $M^{j*} = 0$ or $M^{j*} = M{}_{\rm max}$($j = 2$, 3), the conclusion is invalid.

(ii) $M^{2*}$, $F^{2}$, $D^{2}$, $\Pi_{r}^{2}$, $\Pi_{s}^{2}$, $M^{3*}$, $F^{3}$, $D^{3}$, $\Pi_{r}^{3}$, $\Pi_{s}^{3}$ are high sensitive to *a*, $W$, $b$ and $C$, moderate sensitive to $S_{s}$ and $I_{s}$, low sensitive or insensitive to $A$ and $h_{s}$. On the one hand, the result shows that the change of trade credit period is greatly influenced by increasing demand coefficient $a$, default risk coefficient $b$, wholesale price $W$, and production cost $C$. Hence, the two parties should make joint promotional effort to improve the value of $a$, and to reduce the value of $b$, such that lead to higher the demand and higher the two parties revenue. Meanwhile, the supplier should strive to reduce the production costs $C$ through a variety of efficient measures, or raise the wholesale price $W$. Both of strategies can incentive the supplier to willing to offer a longer trade credit period to raise the profit of the retailer and the supplier. On the other hand, the result also imply that some errors in estimating $A$ and $h_{s}$ may result in little deviation from the optimal results. Hence, in practice, the supplier does not have too high surplus production capacity, and does not need to accurately estimate on the inventory holding cost $h_{s}$.

(iii) For the same conditions, the profits of the retailer, the supplier, and supply chain in a supplier-Stackelberg game are better than the results in Nash game, i.e., $\Pi_{r}^{3} > \Pi_{r}^{2}$, $\Pi_{s}^{3} > \Pi_{s}^{2}$, and $\Pi_{sc}^{3} > \Pi_{sc}^{2}$ in the sensitivity analysis. This is because the optimal trade credit period $M^{3*}$ is greater than $M^{2*}$, such that the market demand from supplier-Stackelberg game is greater than from Nash game, i.e., $D^{3} > D^{2}$. However, we find that the supplier will burden higher default risk in a supplier-Stackelberg game, i.e., $F^{3} > F^{2}$. Likewise, if $M^{j*} = 0$ or $M^{j*} = M{}_{\rm max}$($j = 2$, 3), the conclusion is invalid.

(iv) Under the same conditions, we have ${{\Pi_{r}^{3} } \mathord{\left/ {\vphantom {{\Pi_{r}^{3} } {\Pi_{r}^{0} }}} \right. \kern-0pt} {\Pi_{r}^{0} }} > {{\Pi_{s}^{3} } \mathord{\left/ {\vphantom {{\Pi_{s}^{3} } {\Pi_{s}^{0} }}} \right. \kern-0pt} {\Pi_{s}^{0} }}$ when $M^{3*} > 0$. There are two major reasons can explain the phenomenon. First, the supplier burdens an additional capital opportunity cost, i.e., $CKe^{aM} I_{s} M$. Second, the supplier burdens the default risk of trade credit from the retailer reduces his or her expected net revenue. Moreover, as shown in Table 1, we find that if $0 < M^{3*} < M_{\rm max}$, ${{\Pi_{r}^{3} } \mathord{\left/ {\vphantom {{\Pi_{r}^{3} } {\Pi_{r}^{0} }}} \right. \kern-0pt} {\Pi_{r}^{0} }}$, ${{\Pi_{s}^{3} } \mathord{\left/ {\vphantom {{\Pi_{s}^{3} } {\Pi_{s}^{0} }}} \right. \kern-0pt} {\Pi_{s}^{0} }}$ and ${{\Pi_{sc}^{3} } \mathord{\left/ {\vphantom {{\Pi_{sc}^{3} } {\Pi_{sc}^{0} }}} \right. \kern-0pt} {\Pi_{sc}^{0} }}$ also increase as *a*, $A$ and $W$ increase, and decrease as $b$, $C$, $S_{s}$, $h_{s}$ and $I_{s}$ increase. Additionally, ${{\Pi_{r}^{3} } \mathord{\left/ {\vphantom {{\Pi_{r}^{3} } {\Pi_{r}^{0} }}} \right. \kern-0pt} {\Pi_{r}^{0} }}$, ${{\Pi_{s}^{3} } \mathord{\left/ {\vphantom {{\Pi_{s}^{3} } {\Pi_{s}^{0} }}} \right. \kern-0pt} {\Pi_{s}^{0} }}$ and ${{\Pi_{sc}^{3} } \mathord{\left/ {\vphantom {{\Pi_{sc}^{3} } {\Pi_{sc}^{0} }}} \right. \kern-0pt} {\Pi_{sc}^{0} }}$ is highly sensitive to *a*, $W$, $b$ and $C$, moderately sensitive to $I_{s}$, and has a low sensitive or insensitive to $S_{s}$, $A$ and $h_{s}$.

(v) In most situations, $\Pi_{sc}^{3} > \Pi_{sc}^{1}$, $\Pi_{sc}^{2} > \Pi_{sc}^{1}$, i.e., the total profits of supply chain under the two games are better than the results under centralized decision, only if the optimal trade credit period should not be too short. That is to say, the supply chain’s total profits with longer trade credit period under the two games are both greater than the profit of centralized decision. It indicates that trade credit can be used as coordination parameter.

## Conclusions

How to determinate an optimal trade credit period? This question is gaining more and more attention from researchers. In this paper, we discuss about two retailer–supplier uncooperative replenishment models with default risk under trade credit policy, i.e., a Nash equilibrium model and a supplier-Stackelberg model.

Generally, the main trait of this paper compared to most existing uncooperative inventory model is that the developed model includes the following aspects: (i) Nash equilibrium game and supplier-Stackelberg game; (ii) the results of decentralized decision and centralized decision without trade credit as comparison benchmarks; (iii) the retailer’s capital opportunity cost equal to its opportunity gain; (iv) trade credit period is a decision variable; (v) the demand and default risk both are exponential functions of trade credit period; (vi) lot-for-lot policy; and (vii) the production rate is finite but the replenishment is instantaneous. The major contribution of the paper is that we fully compare between the results of decentralized and centralized decision without trade credit, Nash equilibrium and a supplier-Stackelberg model with trade credit in detail, and obtain some interesting managerial insights and practical implications.

In this paper, we first derive the existence and uniqueness conditions of the optimal solutions for the retailer and the supplier under non-collaborative replenishment policies, i.e., Nash equilibrium game and supplier-Stackelberg game. Moreover, we develop a set of theorems and corollaries to determine the optimal solution and obtain some managerial insights. For instance, a higher value of $a$, $W$, $A$ and a lower value of $b$, $C$, $S_{s}$, $h_{s}$, $I_{s}$ cause a higher value of $M^{{2\bar{*}}}$ and $M^{{3\bar{*}}}$. Finally, we provide an example and sensitivity analysis to illustrate the proposed strategy. Sensitivity analysis reveals that the total profits of supply chain under the two games are better than the results under centralized decision when the optimal trade credit period isn’t too short, also suggests that the size of $M^{j*}$, $F^{j}$, $D^{j}$, $\Pi_{r}^{j}$, and $\Pi_{s}^{j}$ ($j = 2,3$) have a strong relationship with *a*, $W$, $b$ and $C$. In addition, we present other main managerial insights.

The previous results have some practical implications. On the one hand, a supplier may offer a retailer trade credit to expand the demand under certain conditions, especially for the growth phase or launch phase of a product life cycle, even to avoid lasting price competition from competitors. We usually observe that sales volume increases with trade credit period, but production cost decreases with time during the two stages of product life cycle. On the other hand, trade credit is an important financing tool for retailers, especially, the small and micro or starting-up retailer having lack of capital. However, for trade credit of default risk from retailers, the supplier should carefully select good retailers. Moreover, the retailer should set up a fine credit record in the markets, or a long-term relationship with the supplier.

For further research, we may extend the model to allow for other demand functions, such as quadratic trade credit period demand, varying demand both with trade credit period and time, etc. In addition, we may further consider deteriorating items, shortages, environmental impact, warehouse capacity constraint and single supplier/multi-retailer non-coordination and others. Therefore, the effects of all of these additional scenarios may be incorporated in future research.
